# Factors Associated with Anemia among People Living with HIV/AIDS Taking ART in Ethiopia

**DOI:** 10.1155/2019/9614205

**Published:** 2019-03-03

**Authors:** Ketema Bizuwork Gebremedhin, Tadesse Bedada Haye

**Affiliations:** School of Nursing and Midwifery, College of Health Sciences, Addis Ababa University, Addis Ababa, Ethiopia

## Abstract

**Background:**

Globally, anemia, among people living with HIV/AIDS, is a major public health problem. It has a significant effect on the progression of HIV/AIDS to advanced stages and there are a number of factors that often affect anemia. However, there is little insight regarding factors affecting anemia among HIV/AIDS patients in developing countries, including Ethiopia.

**Objective:**

This study aimed at investigating factors affecting anemia among people living with HIV/AIDS taking ART drug at Tikur Anbessa Specialized Hospital, Addis Ababa, Ethiopia.

**Methods:**

A hospital based cross-sectional study design was used to assess factors affecting anemia among people living with HIV/AIDS. Structured checklist was used to gather information from charts of patients selected by simple random sampling method. We analyzed the data to identify factors associated with anemia among people with HIV/AIDS using logistic regression models.

**Results:**

A total of 301 selected charts were reviewed. The median age was 38 ± 10.38. The majority (62.5%) of the patients were taking ZDV-containing ART drug (ZDV/3TC/NVP). The overall anemia prevalence was 34.6%, while about 5%, 15.6%, and 14% of the patients had severe, moderate, and mild prevalence of anemia, respectively. Factors that were found to affect anemia among these patients include gender (OR = 2.26 [95% CI: 1.22, 4.16]), occupation (OR: 0.57 [95%CI: 0.35, 0.92]), WBC count (OR = 2.30 [95% CI: 1.29, 4.09]), platelet count (OR = 2.89 [95% CI: 0.99, 8.41]), nutritional status (OR = 2.05 [95% CI: 0.69, 6.02]), and WHO clinical stage of HIV/AIDS (OR = 3.69 [95% CI: 1.86, 7.31]).

**Conclusions:**

About one in three patients was found to be anemic. Intervention aimed at diagnosing and treating anemia among people living with HIV/AIDS should be considered.

## 1. Introduction

Anemia is a major public health problem, especially in developing countries [1–3]. It has significant consequences on health and social and economic development of individuals [4]. The most common causes of anemia are deficiency of mineral, iron, and vitamin B12 [5], infestation of hookworm, malaria infection, vitamin A deficiency, genetic defects, and chronic infections like TB and HIV [6]. 

Undeniably, anemia is the most important clinical problem seen in people living with HIV/AIDS [7]; its severity increases as CD4 count declines [8] and with progression of the disease HIV/AIDS to advanced stage [9]. In addition, the higher anemia level by itself is also a good opportunity for progression of HIV/AIDS irrespective of CD4 counts level and viral load [10]. Moreover, anemia influences the natural history of the disease HIV/AIDS [11], resulting in the decrease of the survival rate. A number of previous studies from developing countries have demonstrated this evidence [12, 13].

HIV leads to declining the production of cytokine, which ultimately influences hematopoiesis process of the cells, specifically erythropoietin concentration [11]. This, in turn, results in ineffective/decreased production of red blood cells and/or increases their destruction [14]. The most common factors related to the reduced production of red blood cells are acquisition of neoplasm to bone marrow, bone marrow infection due to the HIV itself, administration of myelodepressive chemotherapies like ZDV, and autoantibody proteins produced due to chemotherapies [14, 15]. However, a number of studies revealed that use of HAART reduces anemia among people living with HIV/AIDS [16, 17].

Previous studies conducted in Ethiopia showed that the prevalence of anemia among people living with HIV/AIDS taking ART ranged from 20.9% to 70.1% [14, 18–20]. Factors that are often associated with anemia among people living with HIV/AIDS include ART regimen, presence of opportunistic infections (OIs), residence, gender, marital status, monthly income, educational status, duration of ART taken, history of antituberculosis (TB) drug treatment, advanced stage of the HIV disease, white blood cell (WBC) count < 4,000 cells/*μ*L, CD4+T-lymphocyte count < 200 cells/*μ*L, and platelets count < 200,000 cells/*μ*L [21–23]. However, there is limited insight regarding the prevalence and factors associated with anemia level among people living with HIV/AIDS taking ART drug in Addis Ababa, Ethiopia. The purpose of this study was to estimate the prevalence and examine factors associated with anemia among people living with HIV/AIDS taking ART drug at ART clinic of Tikur Anbessa Specialized Hospital in Addis Ababa, Ethiopia. 

## 2. Methods

### 2.1. Study Design and Settings

A cross-sectional study design was conducted from April 15 to May 15, 2018, at Tikur Anbessa Specialized Hospital in Addis Ababa, Ethiopia. The hospital is among referral and teaching hospitals of the country located in Addis Ababa. The hospital has HIV/AIDS care-providing center with an average daily client flow of 50–60.

### 2.2. Sample Size Determination, Sampling Procedure, and Study Subjects

Sample size was estimated using a single population proportion formula and calculated with the following assumptions: 95% confidence level, 5% margin of error, and 23% expected prevalence of anemia among people living with HIV/AIDS [20]. Given these assumptions and considering a nonresponse rate of 10%, the required sample size was found to be 301. Simple random sampling method was used to select charts to be reviewed among charts of clients who have been followed up during the data collection period on daily basis. Charts of client, with incomplete information, age under 18, pregnant women, postpartum period, and ART inexperienced (those who took ART drug for less than three months), were excluded from the study. The most recent data was taken as information recorded for the study from laboratory investigation sheets and other information sheets.

### 2.3. Study Variables

#### 2.3.1. Anemia Level

The anemia level was determined based on WHO report 2001 and a study conducted on pathogenesis and clinical implications of HIV-related anemia in 2013; the anemia level for nonpregnant women was defined as hemoglobin < 12.0 g/dl: [severe < 7.0 g/dl, moderate = 7–9.9 g/dl, and mild = 10–11.9 g/dl], whereas, for men, it was defined as hemoglobin <13.0: [severe < 9.0 g/dl, moderate = 9–11.9 g/dl, and mild = 12.0–12.9 g/dl] [24, 25].

#### 2.3.2. Sociodemographic and Clinical Characteristics of the Patients

The sociodemographic characteristics identified from the patients' chart include age, gender, residence, religion, ethnic group, marital status, educational status, occupation, and monthly income. Moreover, the clinical and biochemical test characteristics of patients gathered from charts include CD4 count (cells/*μ*L), WBC count (cells/*μ*L), platelet count (cells/*μ*L), nutritional status (BMI) (kg/m^2^), WHO clinical stage of HIV/AIDS, ART regimen, and anti-TB treatment history. The CD4 T-lymphocyte was determined based on WHO (World Health Organization) recommendation of 2007 to commence ART for HIV positive peoples [26]. The WBC and platelet ranges were determined based on the guide of Bacovsky J, blood count interpretation for health personnel [27]. In order to assess nutritional status, patients' body mass index was calculated as a proportion of weight in kg divided by height squared. Then, they were indexed based on body mass index level considered by healthcare practitioners [28]. The clinical stage of HIV/AIDS was used based on the WHO clinical staging classification criteria [26]. The ART regimen was determined based on whether the study participants were taking ZDV-containing ART drug or not. Similarly, treatment history of the client was assessed for being treated for TB or not.  Table 1 summarizes the cut-off points used for blood cell levels.

### 2.4. Data Collection Instrument, Data Collection Processing, and Quality Control

The questionnaire (checklist) was developed after reviewing literatures and WHO guidelines. The checklist comprised sociodemographic and clinical characteristics: age, gender, residence, religion, ethnic group, marital status, educational status, occupation, monthly income, and clinical characteristics: CD4 T-lymphocyte, hemoglobin, WBC, platelet count level, BMI level, WHO clinical stage of HIV/AIDS, ART regimen, and history to treatment for TB. Before the actual data collection date, the questionnaire was pretested on 5% of charts of the study population. Then, necessary modification was done based on its analysis. The study questionnaire was prepared in English. A graduate class nurse student collected the data. Principal orientation was given for data collector prior to the data collection date on how to collect the data and make them familiar with the data collection questionnaire. The completeness of each questionnaire was checked on daily basis.

### 2.5. Data Analysis

Data was entered using EpiData version 3.1 statistical package software. After thorough cleaning, the data was exported to SPSS version 22 statistical package software for further analysis. Descriptive statistics such as mean, standard deviations (SD), frequency, and percentage were used to describe the variables of the study. In order to describe factors associated with anemia among people living with HIV/AIDS, unadjusted and adjusted odds ratios with their 95% CI were calculated using logistic regression models.

### 2.6. Ethical Consideration

The ethical clearance letter was obtained from ethical clearance committee of Addis Ababa University College of Health Sciences. After we offered the letter to the head department of ART clinic, the purpose of the study was clearly stated to the healthcare professionals working in the ART clinic of the Hospital.

## 3. Results

A total of 301 charts were reviewed. The median age was 38±10.38. The majority (62.1%) of the study participants were female. About half (50.5%) of the study participants were unemployed. More than three-fourths (90%) of the study participants were from urban residential area. About half (49.8%) of the study participants were married.  Table 2 summarizes the details of the characteristics of study participants.

### 3.1. The Clinical and Biochemical Tests Characteristics

The medians (±SD) of CD4, WBC, and platelet counts were 332** ±** 195.67, 5490** ±** 4015, and 287000 ± 97499.53, respectively. The majority (77.1%, 68.4%, and 93.4%) of the study participant's CD4, WBC, and platelet count were** ≥** 200,** ≥** 4500, and** ≥** 150,000 cells/*μ*L, respectively. Regarding the study participant's nutritional status, 61.5%, 20.6%, 12%, and 6% of the study participants were found to be of normal weight, overweight, underweight, and obese in their nutritional status, respectively. Further, almost three-fourths of the study participants were taking ZDV-containing ART drug (ZDV/3TC/NVP) (Table 3).

### 3.2. Prevalence of Anemia

**Figure 1:**
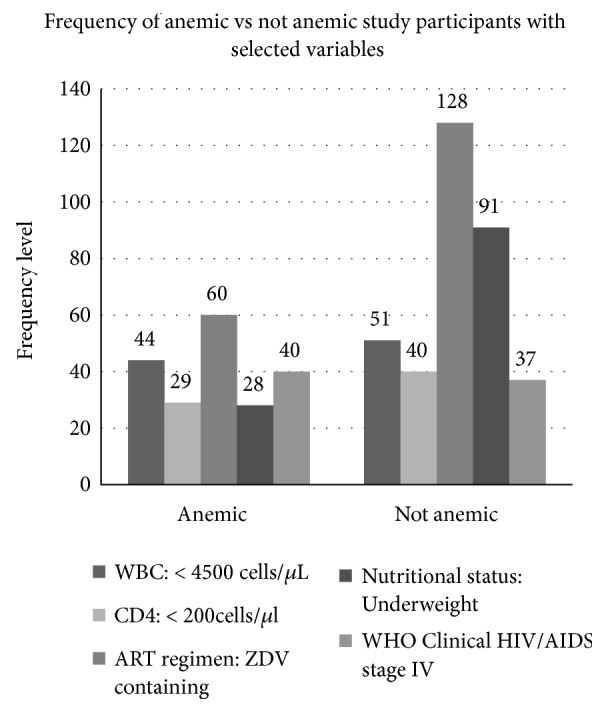
Frequency of anemic versus not anemic study participants with selected variables.

The prevalence of anemia was 34.6%. Among the overall anemic study participants, 5%, 15.6%, and 14% had severe, moderate, and mild level of anemia, respectively. A significant number (48) of study participants were from the age group of 30-39. A momentous number (75 and 60) of anemic study participants had CD4 T-lymphocyte and WBC count ≥ 200 and ≥ 4500 cells/*μ*L, respectively. A noteworthy number (40) of anemic study participants were found in stage IV of WHO clinical stage of HIV/AIDS. Regarding ART regimen and anti-TB drug exposure history, a considerable number (60 and 36) of anemic study participants were taking ZDV-containing ART drug (ZDV/3TC/NVP) and had history of anti-TB drug treatment exposure, respectively (Table 3). Among a total of 95 study participants whose WBC count level was < 4500 cells/*μ*L, 44 were anemic, while the remaining 51 were not anemic. Moreover, among a total of 77 study participants who were found in stage IV of WHO clinical staging of HIV/AIDS, 40 were anemic, while the remaining 37 were not. Further, among a total of 188 study participants who were taking ZDV-containing ART drug (ZDV/3TC/NVP), a considerable number (60) were anemic, while the remaining 128 were not (Figure 1).

### 3.3. Factors Associated with Anemia

The unadjusted logistic regression analysis revealed that sociodemographic characteristics, gender (*χ*^*2*^ = 6.89,* p *< 0.010) and occupation (*χ*^*2*^ = 5.34,* p *< 0.022), were significantly associated with anemia (Table 2). Regarding clinical characteristics, WBC count (*χ*^*2*^ = 8.33,* p* < 004), platelet count (*χ*^*2*^ = 5.78,* p* < 0.18), nutritional status (*χ*^*2*^ = 23.45,* p* < 0.001), and WHO clinical HIV/AIDS stage (*χ*^*2*^ = 16.72,* p* < 0.001,) were significantly associated with anemia (Table 3). The adjusted logistic regression analysis showed that gender (OR = 2.26 [95% CI = 1.22, 4.16],* P* < 0.009), WBC count (OR = 2.30 [95% CI = 1.29, 4.09],* p *< 0.005), platelet count (OR = 2.89 [95% CI = 0.99, 8.41],* p* < 0.051), nutritional status (OR = 2.05 [95% CI = 0.69, 6.02],* p* < 0.001), and WHO clinical HIV stage (OR = 3.69 [95% CI = 1.86, 7.31],* p* < 0.003) were among the different factors associated with anemia among people living with HIV/AIDS (Table 4).

## 4. Discussion

This study assessed the prevalence and factors associated with anemia among people living with HIV/AIDS taking ART for at least three months in Tikur Anbessa Specialized Hospital in Addis Ababa, Ethiopia. It is clear that anemia is a common medical problem affecting people living with HIV/AIDS by complicating the pathogenesis of the disease [9, 19, 25]. Using anemia demarcation level < 13 g/dl [24, 25], our study found that the prevalence of anemia was 34.6%. This figure is lower when compared to previous studies completed in Ethiopia (the prevalence ranged from 43% to 70.1%) [9, 14, 22], Iran, Nigeria, China, and Nepal, ranging between 51.5 and 71% [13, 29–33]. However, the prevalence of anemia reported in this study was higher than a number of studies conducted in different parts of Ethiopia ranging between 20.8 and 23.1% [18, 20, 21, 34–37] and South Africa (25.8%) [10]. Moreover, this study revealed that 5%, 15.6%, and 14% of the study participants had severe, moderate, and mild level of anemia, respectively. This finding is in line for the severe level of anemia while it is lower for moderate and mild level of anemia in another study from Ethiopia [36]. The possible reason for the difference observed in prevalence of anemia may be due to the difference in socioeconomic level, difference in study participants' WHO clinical HIV/AIDS staging level, difference in ART regimen taking, presence/absence and/or level of opportunistic infections, lack of awareness about appropriate nutrition, and inclusion and/or exclusion criteria in recruiting study participants.

The finding of this study indicates that almost one-third of the study participants were anemic, which is a concern, as untreated anemia in people living with HIV/AIDS and/or taking ART is associated with pathophysiologic changes and progression of the disease to advanced stage and finally may result in death of the individual [25]. Thus, there should be an intervention that has an ultimate effect in reducing mortality related to complications of anemia among people living with HIV/AIDS and/or taking ART drug.

Factors associated with anemia include gender, occupation, WBC count, platelet count, nutritional status, and WHO clinical staging level. These findings are partly in line with similar previous study findings [9, 11, 18, 21]. According to the findings of this study, females were more likely to be anemic compared to males. This finding is in line with studies from Nigeria and South Africa [10, 29]. However, the unemployed study participants were less likely to be anemic compared to the employed study participants. Similarly, younger study participants were found to be less likely to be anemic compared to the elders. This finding is in line with studies from Ethiopia [10, 14]. This may be due to the fact that the younger study participants' cell proliferating organs have more competent proliferation capacity of red blood cell and the participants themselves are more aware about benefit of consuming more nutritional diet and/or have a better socioeconomic and educational status.

Moreover, we found that underweight study participants were more likely to be anemic, while those with normal weight were less likely to be anemic. This finding is consistent with previous studies conducted in developing countries [10, 12, 38]. This may be due to the fact that the underweight individuals are less aware about the benefit of appropriate nutrition consumption and/or have a less socioeconomic and educational condition or may have less adherence to ART drug therapy. Further, those study participants taking ZDV-containing ART drug were found more likely to be anemic compared to those who were not. This finding is in line with similar studies from Ethiopia [11, 13, 20, 35]. This is usually due to the side effect of the ART drug ZDV resulting in manifestation of granulocytopenia, anemia, malaise, fatigue, and other gastrointestinal symptoms [39].

Bearing in mind the effect of anemia and its significant prevalence, it is a great disquiet that more than one-third of the study participants were anemic, while appropriate treatment options are available and the study participants were on ART drug for at least three months, which have an effect to combat the pathogenesis of the disease. Therefore, based on the findings of this study, the following interventions are suggested to detect and combat anemia among people living with HIV/AIDS taking ART drug.* (1)* Monitor hemoglobin level on each of the follow-up periods by focusing on those people taking ART drug regimen containing ZDV and take appropriate action to combat the anemia if anemia is detected. This is because the pathogenesis of anemia is worrisome and ZDV by itself has an effect on reducing proliferation of red blood cells.* (2)* Monitor adherence level of the people living with HIV/AIDS taking ART drug to their therapy and motivate, encourage, and advise them to adhere to the ART drug therapy consistently to gain its therapeutic effect. This is because ART drug has an effect on combating anemia among people living with HIV/AIDS taking ART drug.* (3)* Monitor and take quick appropriate action for drug-drug interaction and magnified side effects of each drug which may occur in people living with HIV/AIDS taking ART drug. This is because drug-drug interaction and side effects of some drugs by themselves interfere with reduced proliferation of granulocytes and contribute to raising immature destruction of cells.* (4)* Increase awareness of people living with HIV/AIDS taking ART drug about the benefit of adhering to consumption of nutritional diet consistently. This is because consuming appropriate diet helps the body in proliferating enough amounts of red blood cells and other granulocytes. This study has limitations. Firstly, it is a cross-sectional study that has chicken egg dilemma in identifying causal relationship. Secondly, the study excludes people living with HIV/AIDS who are naïve to ART drug. Thus, it is recommended that future study considers longitudinal study by considering ART-naïve people living with HIV/AIDS by determining the severity of anemia.

*In conclusion,* it is important to alleviate anemia among people living with HIV/AID taking ART drug, as the presence of anemia among people living with HIV/AIDS fastens the progression of the disease to the advanced stages. The suggested intervention by this study focused at improving nutritional status of people living with HIV/AIDS taking ART drug through enhancing awareness of benefit of consuming balanced diet and monitoring and managing granulocytes level appropriately, which increases survival rate; it is best to monitor granulocytes like CD4 count, platelet and WBC count, and hemoglobin level more frequently. It is also good to focus on sociodemographic characteristics like gender and occupation of the client, which have an ultimate effect in affecting level of anemia among people living with HIV/AIDS.

## Figures and Tables

**Table 1:** Cut-off point for blood cells considered in the study.

Type of blood cell	Cut-off point	Remark

WBC	≥ 4.5 × 10^3^ cells/*μ*L	
	< 4.5 × 10^3^ cells/*μ*L	Below normal
Platelet	≥ 150 x 10^3^ cells/*μ*L	
	*<* 150 × 10^3^ cells/*μ*L	Below normal
CD_4_	≥ 200 cells/*μ*L	Commenced for ART
	< 200 cells/*μ*L	
BMI	< 18.5 kg/m^2^	Underweight
	18.5 – 24.9 kg/m^2^	Normal
	25 – 29.9kg/m^2^	Overweight
	≥ 30kg/m^2^	Obese

*Note. *CD4 = cluster of differentiation 4, WBC = white blood cells, BMI = body mass index, *μ*L = microliter.

**Table 2:** Sociodemographic characteristics and their association with anemia under unadjusted logistic regression analysis (n = 301).

Sociodemographic variables	Frequency (%)	Anemia status		OR (95%CI)	Χ^2^	P value
		Anemic frequency (%)	Not anemic frequency (%)			
		104(34.6)	197(65.4)			

Age in complete years (median age = 38 ± 10.38)					4.56	0.11
18 - 29	53(17.6)	20	33	0.64[0.33, 1.26]		
30 - 39	119(39.5)	48	71	0.57[0.34, 0.97]		
≥ 40	129(42.9)	36	93	∗∗		
Gender					6.89	0.01∗
Female	187(62.1)	75	112	1.96[1.18, 3.28]		
Male	114(37.9)	29	85	∗∗		
Residence					0.43	0.51
Urban	271(90)	92	179	1.30[0.60, 2.81]		
Rural	30(10)	12	18	∗∗		
Religion					0.59	0.44
Christian	261(86.7)	88	173	1.31[0.66, 2.59]		
Muslim	40(13.3)	16	24	∗∗		
Ethnic group					0.19	0.91
Amhara	114(37.9)	40	74	1.07[0.54, 2.11]		
Oromo	135(44.9)	45	90	1.15[0.59, 2.25]		
Other	52(17.3)	19	33	∗∗		
Marital status					0.86	0.35
Married	150(49.8)	48	102	1.25[0.78, 2.02]		
Unmarried	151(50.2)	56	95			
Educational status					3.00	0.60
Not read and write	52(17.3)	19	33	0.52[0.21, 1.33]		
Read and write	63(20.9)	23	40	0.52[0.21, 1.29]		
Primary school	87(28.9)	30	57	0.57[0.24, 1.36]		
Secondary school	60(19.9)	23	37	0.48[0.20, 1.20]		
College and above	39(13.0)	9	30	∗∗		
Occupation					5.34	0.02∗
Unemployed	152(50.5)	43	109	0.57[0.35, 0.92]		
Employed	149(49.5)	61	88	∗∗		

*Note. Religion:* Christian includes orthodox, protestant, and catholic. *Ethnic group:* other includes Gurage and Tigre. *Marital status:* Unmarried includes living together, widowed, single, and divorced. *Occupation:unemployed *includes merchant, housewife, student, farmer, and daily laborer. Employed includes private and governmental employees.

Statistical significance at 95% CI, P < 0.05; ∗∗ reference; ∗ statistically significant.

**Table 3:** Clinical characteristics and their association with anemia under unadjusted logistic regression analysis (n = 301).

Background characteristics	Frequency (%)	Anemia status		OR (95%CI)	Χ^2^	P value

		Anemic frequency (%)	Not anemic frequency (%)			
		104(34.6)	197(65.4)			

CD4 count level (cells/***μ***L) (median CD4 count = 332 ± 195.67)					2.17	0.14
≥ 200	232(77.1)	75	157	1.52[0.87, 2.64]		
<200	69(22.9)	29	40	∗∗		
WBC count level (cells/***μ***L) (median WBC count = 5490 ± 4013.00)					8.33	0.00∗
≥ 4500	206(68.4)	60	146	2.10[1.27, 3.47]		
<4500	95(31.6)	44	51	∗∗		
Platelet count level (cells/***μ***L) (median platelet count = 287000 ± 97499.53)					5.78	0.01∗
≥ 150, 000	281(93.4)	92	189	3.08[1.22, 7.80]		
<150,000	20(6.6)	12	8			
BMI (kg/m^2^)					23.45	0.00∗
Underweight	36(12)	24	125	1.67[0.63, 4.44]		
Normal	185(61.5)	60	12	0.40[0.13, 1.28]		
Overweight	62(20.6)	12	50	3.33[1.08, 10.25]		
Obese	18(6)	8	10	∗∗		
WHO Clinical HIV/AIDS stage					16.72	0.00∗
Stage I	119(39.5)	28	91	3.51[1.90, 6.50]		
Stage II	61(20.3)	20	41	2.22[1.10, 4.45]		
Stage III	44(14.6)	16	28	1.89[0.89, 4.44]		
Stage IV	77(25.6)	40	37	∗∗		
ART regimen					1.53	0.22
ZDV containing	188(62.5)	60	128	1.36[0.84, 2.21]		
Not ZDV containing	113(37.5)	44	69	∗∗		
Anti-Tb treatment history					0.61	0.44
Yes	104(34.6)	36	65	0.82[0.50, 1.35]		
No	197(65.4)	65	132	∗∗		

Note: CD4 = cluster of differentiation 4, WBC = white blood cells, BMI = body mass index, ZDV = zidovudine, *μ*L = microliter.

∗∗ reference; ∗ statistically significant.

**Table 4:** Logistic regression analysis showing the association between patient characteristics and anemia among people living with HIV/AIDS (n = 301).

Variables	*β*	SE	Wald *χ*^2^	OR (95%CI)	P value

Gender	0.81	0.31	6.80	2.26[1.22, 4.16]	0.01
WBC count	0.83	0.29	7.99	2.30[1.29, 4.09]	0.01
Platelet count	1.06	0.55	3.80	2.89[0.99, 8.41]	0.05
Nutritional stats	0.72	0.55	17.92	2.05[0.69, 6.02]	0.00
WHO HIV/AIDS clinical staging	1.31	0.35	14.31	3.69[1.86, 7.31]	0.00

*Note.* HIV/AIDS = Human Immune Deficiency Virus/Acquired Immune Deficiency Syndrome, SE = standard error, WBC = white blood cells, OR = odds ratio, CI = confidence interval.

## Data Availability

The data used to support the findings of this study are available from the corresponding author upon request.
